# Opportunities of Genomics for the Use of Semen Cryo-Conserved in Gene Banks

**DOI:** 10.3389/fgene.2022.907411

**Published:** 2022-07-14

**Authors:** J. Kor Oldenbroek, Jack J. Windig

**Affiliations:** Centre for Genetic Resources, The Netherlands and Animal Breeding and Genomics, Wageningen University & Research, Netherlands, Netherlands

**Keywords:** genomics, genetic diversity, priorization, optimization, introgression, selection, drift

## Abstract

Shortly after the introduction of cryo-conserved semen in the main farm animal species, gene banks were founded. Safeguarding farm animal genetic diversity for future use was and is the main objective. A sampling of sires was based on their pedigree and phenotypic information. Nowadays, DNA information from cryo-conserved sires and from animals in the living populations has become available. The combination of their DNA information can be used to realize three opportunities: 1) to make the gene bank a more complete archive of genetic diversity, 2) to determine the history of the genetic diversity from the living populations, and 3) to improve the performance and genetic diversity of living populations. These three opportunities for the use of gene bank sires in the genomic era are outlined in this study, and relevant recent literature is summarized to illustrate the great value of a gene bank as an archive of genetic diversity.

## Introduction

### Conservation of Genetic Diversity in Gene Banks

Genetic diversity is an important characteristic of a population of animals. It creates the opportunity for artificial selection to improve desired traits of the animals. Genetic diversity is important in natural and captive populations because it facilitates their adaptation to a wide variety of environments. Genetic diversity is influenced by genetic drift, selection, migration, and mutation. The loss of genetic diversity within breeds, resulting in inbreeding, is mainly at stake in populations under intense selection for a few traits and in small populations with a high genetic relationship between the animals. The conservation of the genetic diversity between breeds is also relevant because recently many breeds were set aside from the mainstream production chains. These rare breeds may be a source of unique genetic diversity if they still have a sufficient effective population size (Leroy et al., 2015).

Concern about the loss of genetic diversity in farm animals has become widespread, for example, by the activities of the FAO (Oldenbroek, 2007). This loss can effectively be prevented, among other measures, by the storage of frozen semen or embryos (Smith, 1984). In the 1940’s; of the past century, artificial insemination techniques were developed. The main driver in cattle was the existence of venereal diseases transferred by natural mating, which caused infertility. In the 1950’s, cryo-conservation of semen was developed to facilitate the logistics and a wider use of sires not hindered by the short longevity of fresh semen. Already at the start of artificial insemination in cattle with frozen semen, Swedish AI-studs started with the long-term storage of cryo-conserved semen from each bull used for breeding (Oldenbroek, 1999).

Smith (1984) stated that “the possible returns from retaining genetic diversity may be large, while the costs for cryo-conservation in a gene bank, by comparison, are trivially small on a national basis.” In his view, any breed at risk should be cryo-conserved. However, he predicted that “the continuous genetic improvement in current stocks may make it increasingly difficult for unimproved conserved stocks to compete, unless there are reversals in breeding goals or drastic changes in husbandry conditions.” Despite the fact that breeds, not used by mainstream breeding programs, are presently kept alive in small numbers by motivated farmers, their existence and their genetic diversity are not safeguarded in the future by live-conservation only. These breeds are often less productive and generate less income and are kept by hobby-farmers or older professional farmers, often without a successor for their farm. Therefore, the integration of cryo-conservation of genetic diversity in a gene bank with the conservation of genetic diversity in live populations is the most powerful conservation strategy (Oldenbroek, 2007).

An example of a gene bank in progress is described by Blackburn et al. (2019). The US gene bank already contains more than one million samples from over 55,000 animals from 165 livestock and poultry breeds. The collection was developed to safeguard the genetic diversity of species and breeds important for livestock production. The oldest samples are from animals that lived 60 years ago. About 50% of the collection comprises rare breeds, with less than 1,000 animals. Their collection completeness is 45%. The completeness is calculated as a percentage of the target goal, which combines the germplasm quantity and the minimum number of animals to reconstitute a breed with an effective population size of 50. The larger populations are more complete, up to 98%. Gene bank collections are used indeed. Samples from over 6,000 animals in the collection have been used for adding diversity to breeds, genomic evaluation, reconstituting populations, or various research projects. Especially for the rare breeds, confronted with an array of obstacles not at stake in the larger populations, the gene bank is considered in the US to be the best security for the U.S. livestock sector.

### Opportunities for Genomics to Use Conserved Genetics

In the era of genomics, the management of gene banks can take their decisions for sampling and for use of the donors of semen based on genomic criteria. In the past, the genetic criteria could only be based on the history of breeds and on pedigree analysis (Passemard et al., 2018). “Measures of genetic relationships and inbreeding based on pedigrees are expectations, while molecular genetic estimates of inbreeding are the particular realizations of such expectations” (Fernández and Bennewitz, 2017). This is the reason that genomic-based measures of genetic relationships between breeds measure the genetic distances among breeds more accurately. The cost of generating genomic data on a large scale has decreased sharply in recent years. For these reasons, genomic information has become the standard for choices to be made in the conservation of farm animal genetic diversity (Oldenbroek, 2017).

Nowadays, when gene bank collections become more or less complete, other questions become relevant (Passemard et al., 2018): what are the useful additions from the live populations; how to optimize the collection; and last but not least, how can these collections serve the breeding programs for the live, small, and even mainstream populations? What are the opportunities of genomic techniques in this respect? The DNA information of cryo-conserved sires and from animals in the living populations can be combined. This combination can be used 1) to make the gene bank a more complete archive of genetic diversity, 2) to determine the genetic background of the living populations, and 3) to improve the performance and genetic diversity of living populations. These three opportunities for the use of gene bank sires in the genomic era will be outlined in this study. Relevant recent literature (from 2016 onwards) will be summarized to illustrate the great value of a gene bank as an archive of genetic diversity.

## Prioritization and Optimization Processes in Gene Bank Collections Based on Between and Within Breed Genetic Diversity

### Methods to Prioritize Breeds or Lines for Conservation in Gene Banks

Conservation of breeds in gene banks requires sampling and freezing of semen and other genetic material. It is labor-intensive when the sires are not used in regular breeding programs. The sampling of breeds and male animals within breeds is based on choices for which genomic similarities between breeds and animals might be decisive. Unique haplotypes and alleles may also influence such decisions. Fernández and Bennewitz (2017) described three methods useful to prioritize breeds with SNPs or WGS data: 1) the Weitzman diversity based on genetic distances and 2) the core set diversity based on genetic similarities, within and across breeds. 3) the so-called cluster analysis. The cluster approach attempts to detect an unknown number of groups (clusters) in the whole population. In this method, the genotypic data determine the structure of the clusters based on similarities and differences in genetic markers. The distance between the centers of the clusters indicates the genetic distances between breeds. The circumference of the clusters indicates the within-breed genetic diversity. In all these methods, population size plays a role. Rare breeds often have a small effective size and little within breed diversity, but they can have a large distance from other breeds. In the core set method, the little within-breed diversity is weighted against the large between-breed diversity. The outcome determines the genetic contribution of a breed to the genetic diversity of the species. In the Weitzman method, only the genetic distances to the other breeds are decisive in this respect.

The core set diversity method can be extended to the “safe set +1” approach (Eding et al., 2002). The start is the detection of the group of breeds not at risk of extinction or the breeds that are expected not to become extinct, and then the diversity stored in that set of breeds is calculated. The breeds outside this set are added one by one, and the increase in diversity for each breed is calculated. The breeds giving the highest increase in diversity get the highest priority for conservation.

Hulsegge et al. (2019a) used a combination of core set and cluster analysis to study the relationship between the different Landrace pig lines conserved in the Dutch gene bank and their genetic relationship with the present population of Landrace pigs from Topigs Norsvin. Two clusters were identified in the conserved lines: the Norwegian/Finnish Landrace lines and the Dutch Landrace lines. These lines were bred in the past by different companies. With the gene bank samples, it was possible to assess the effect of a series of mergers of breeding companies in which lines were set aside or were used to breed the present Dutch Landrace line. Structure analysis revealed that all Landrace lines in the gene bank had a unique diversity and contributed almost equally to the present Dutch Landrace line. The core set method revealed that the genetic diversity level of the current Dutch Landrace breed was 0.89, while from the whole set, it was 0.99. Thus, a large quantity of the genetic diversity of the conserved Dutch Landrace lines in the gene bank is still present in the Dutch Landrace line of Topigs Norsvin. But, the gene bank lines harbor 10 per cent of the total genetic diversity not present in this current Dutch Landrace line.

Huson et al. (2020) compared the genetic variation of 49 heavily used Jersey bulls from the Island of Jersey to the genetic variation of 47 non-Island Jersey bulls and cows, mainly from the U.S, using a 777 K SNP chip. The Island of Jersey’s population has been isolated for a long time from Jersey cattle elsewhere. Principal component analysis demonstrated that the two populations clearly differed but clustered together when Guernsey and Holstein cattle were incorporated in the analysis. The two Jersey populations demonstrated similar inbreeding levels despite large differences in population size and gene flow and slightly higher estimates of inbreeding parameters compared to the Holstein and Guernsey populations. This study provided an overview of how genetic variation in the Jersey breed was shaped, which can serve as a reference for future management of its genetic diversity.

Signer-Hasler et al. (2017) compared original local Swiss cattle breeds (Original Braunvieh (OB), Simmental (SI), Eringer (ER), and Evolèner (EV)) to more widely used breeds (Brown Swiss, Braunvieh Holstein, Red Holstein, and Swiss Fleckvieh). This was carried out with the genotypes of 9,214 cryo-conserved sires. They demonstrated low levels of genomic inbreeding and high levels of genetic diversity in the original Swiss cattle populations ER, OB, and SI compared to the other breeds and explained it by a greater use of natural service in the original Swiss breeds. The EV population had a high level of genomic inbreeding because it is regionally restricted with a low number of herd-book cows.

In several other studies, the analyzed DNA data originated partly from cryo-conserved males and partly from living animals. For example, Upadhyay et al. (2019) analyzed a mixture of gene bank bulls and live animals of nine native Swedish cattle breeds and described the genetic history and the population structure of the Swedish cattle breeds. They could detect clusters of breeds based on a common history and lines within breeds and detect differences in genetic diversity within breeds. Gautason et al. (2020) studied, partly with cryo-conserved bulls, the genetic position of Icelandic cattle, bred in isolation over 1,000 years, in the pool of Nordic and West-European breeds. They concluded that the Icelandic cattle have the highest relationship with the Finncattle breeds (Eastern, Northern, and Western Finncattle), and the Swedish Mountain cattle. Due to the long period of isolation, Islandic cattle is highly genetically distinct from the other Nordic and West-European breeds. Schmidtmann et al. (2021) analyzed ten cattle breeds belonging to the group of red dairy breeds in Northern Europe and originating from Germany, the Netherlands, and Denmark. The genomic composition was analyzed with 36195 SNP’s. Genetic relationships and shared ancestries differed between the breeds. Gene flow from the Red Holstein breed to two German Angler breeds was clearly established.

### Methods Useful for the Optimization of Gene Bank Collections

The characterization of samples by marker genotypes (SNP) or by Whole Genome Sequencing (WGS) of germplasm can give important additional information to the existing pedigree and phenotype-based information (Berg and Windig, 2017). The selection of animals in living populations for sampling of their semen, oocytes, or embryos for cryo-conservation can be performed more accurately with genomic information. More accurately, in the sense that the selected animals increase the genetic diversity of the samples of the species already conserved. Also, it guarantees that specific alleles or haplotypes are included in the cryo-collection. Software to estimate optimal contribution, including constraints for breeding value or genetic diversity, is available, for example,

Gencont, http://www.genebankdata.cgn.wur.nl/gencont/gencont.html.

EVA, http://www.nordgen.org, and

MateSel, http://matesel.une.edu.au.

Doekes (2020) stated that “a common strategy of national gene banks is to conserve all national breeds.” But funds are usually restricted, and choices between breeds often have to be made. This may depend on which other breeds are already conserved. Due to historical events such as the time of separation or migration, closely related breeds share more diversity than less related breeds. The three main criteria for deciding which breeds should be conserved in a gene bank are genetic diversity, utility, and extinction probability (Bennewitz et al., 2007). When breeds are genotyped, we obtain more accurate estimates of the within-breed diversity. Bennewitz et al. (2007) outlined a strategy that maximizes utility in the selection of breeds for conservation. This utility combines the within breed diversity, the specific traits and the value of sustainable utilization of the breed, for example, for economic, cultural, and nature conservancy applications.

The most important aim of a breed’s conservation is to establish a core collection per breed that is sufficiently large enough to reconstitute that breed when it becomes extinct. Within breeds, the main goal is to maximize genetic diversity in the core collection. For a core collection, genomics can be used to select the animals from the living population. The actual number of samples needed in such a core collection, the strategic size consisting of the number of sires per breed and the number of samples per sire, is determined by the objectives of the collection. For the restoration of a population, the number of samples partly depends on the insemination success of the cryo-conserved semen (see FAO (2012) for more details). An optimization process for the core collection may result in moving samples from individual animals from the core collection to the working collection. Semen from the working collection can be distributed upon request for research or support of the live population.

For the selection of animals for the core collection of breeds, SNP or WGS data can be used in optimal contribution methods. SNP data have the disadvantage of ascertainment bias because they are often developed for the use in mainstream breeds. WGS also detects minor alleles present in rare breeds. Eynard et al. (2016) concluded that the amount of diversity conserved is approximately the same using SNP or WGS data in an optimal contribution method. But using WGS data, the loss in small minor alleles is much less than based on data from SNP chips.

Hulsegge et al. (2016) used the maximum diversity strategy to determine the contribution of the Dutch Red and White Friesian (DFR) cattle (gene bank samples and samples from living females) and the contribution of different lines within this breed to the national cattle gene pool. With the use of the principle component analysis, genomic relationship measurements, and the core set plus one approach, it could be concluded that: 1) the DFR breed has a small but unique contribution to the Dutch cattle gene pool; 2) it is closely related to the Black and White Dutch Friesian breed; 3) of the seven lines that can be distinguished within the DFR breed, only two lines are clearly separate. 4) one of the separate lines comprises unique diversity not present within the DFR breed nor in the rest of the Dutch cattle gene pool and 5) the second separate line comprises unique diversity for the DFR breed but its genetic diversity overlaps with that of the Holstein Friesian breed, due to the use of a few Holstein Friesian bulls in the past. Thus both the population structure of a breed and its relationship with other breeds should be taken into account in the conservation decisions for a breed.

Van Breukelen et al. (2019) characterized the genomic diversity in the gene bank for Dutch native cattle breeds with SNP data. The data set consisted of 715 bulls from seven native breeds and 165 Holstein Friesian bulls and was used to calculate genetic similarities. With optimal contribution selection, core sets of bulls were established with a minimized similarity. The gene bank was composed in the past based on pedigree information. This led to a partial optimization of semen collection. The mean similarity within breeds based on the number of straws conserved per bull was 0.32%–1.49% lower than in the case where each bull would have contributed equally. Mean similarity could be further reduced within core sets by 0.34%–2.79% using OCS.

Engelsma et al. (2014) determined the impact of conserving a specific allele in a cryo-collection. That might be a unique allele with a positive effect on the performance or an allele responsible for a genetic defect. The more selection pressure is on a specific allele, the less diversity will be conserved across the genome. This method is not attractive because genetic diversity around the targeted locus will be lost.

In conclusion, for the prioritization of breeds and lines for conservation in gene banks and for the optimization of gene bank collections, several genomic methods were developed and approved to be effective, for example, the core set diversity approach, cluster analysis, and optimum contribution selection. To obtain a complete archive of the genetic diversity of a species within a breed, the analyzed genomic data should be as accurate as possible. Whole-genome sequences are to be preferred over SNPs because WGS data give information on rare variants of alleles and are free of ascertainment bias.

## The Determination of the History of the Genetic Diversity of the Living Populations

In the history of populations, selection plays an important role in several ways. Natural selection results in changes in allele frequencies that enable the adaptation of the animals to current environments. Artificial selection within breeding programs influences the allele frequencies at QTL, leading to desired performance traits. Gene banks are reservoirs to detect the selection of breeds over recent generations by calculating the changes in allele frequencies (Boitard et al., 2021). In an ideal breeding program in full control, all sires (and females) contribute equally to the next generation. But in less controlled breeding programs (e.g., cattle and companion animals), genetic drift is a real risk. In these programs, an intense selection for performance often causes the popular sire effect (Wellmann and Bennewitz, 2019). This effect may lead to the loss of part of the genomes of founder animals over generations that may be missed in the present live populations. Then, gene bank sires might be of value for the live populations when they contain parts of the genome of the lost founders. Their use can re-introduce these parts of the genome and increase the genetic diversity in the live population. A comparison of the live and cryo-bank populations can indicate which part of the genome in the live population is missing and can be re-introduced through the use of gene bank sires with a focus on SNPs, WGS, and minor alleles. In the history of populations, often selection and genetic drift are simultaneously at stake, both resulting in inbreeding and the loss of genetic diversity. The effects of selection and genetic drift are often confounded and cannot easily be untangled.

### Genetic Diversity That Might Be Lost Without Conservation Efforts

Doekes et al. (2018a) assessed the effects on genetic diversity and genetic merit by using cryo-conserved bulls born before 2015 in addition to bulls born between 2010 and 2015. Optimal contribution selection was performed to minimizing mean relatedness (thus maximize diversity) or to maximize genetic merit but with a restriction on relatedness. They concluded that the additional merit from cryo-bank bulls could be considerable when 1) the relative emphasis on diversity was higher, or 2) the index under selection changed. The additional merit of using the cryo-bank bulls was relatively low for the total merit index currently in use but higher (in ascending order) for the sub-indices production, udder health, and fertility. They concluded that: “anticipating changes in breeding goals in the future, the germplasm collection is a valuable resource for commercial breeding populations.”

WGS data detect all genetic variants, including those with a low Minor Allele Frequency (MAF), which are largely absent from Single Nucleotide Polymorphism (SNP) chips. Therefore, WGS data are expected to measure more accurately the genetic relationships in populations. Eynard et al. (2015) compared the effects of using pedigree, SNP or WGS data of 118 Holstein bulls for the prioritization of animals for conservation. The benefit of using WGS was small for common variants, but considerable for variants with a MAF below 5%. Eynard et al. (2016) also investigated the effect of optimal contribution selection based on either data from a 50 k SNP chip or based on WGS data in a population of 277 bulls from the 1,000 bull genome project. Selection with a lot of emphasis on genetic improvement gave a high risk of loss, especially for the rare variants in the population. This risk was lower when the selection was based on genetic relationships determined with WGS data.

Hulsegge et al. (2022) investigated changes in genetic diversity in Dutch Friesian (DF) cattle. This breed is one of the founding breeds of Holstein cattle, but the two breeds have been bred separately for over 100 years. In the 1970’s and 1980’s, Holstein cattle largely replaced DF cattle, and the latter decreased sharply in numbers. Genetic diversity was compared with WSG data between a group of cryo-conserved historic DF sires (hDF) from about 40 years ago, a group of recently used DF bulls (rDF) and a group of recently used HF bulls, respectively. A large overlap of genetic diversity exists between the three groups due to their common history. However, each of the three groups has a number of group-specific SNPs, and the two DF groups are genetically clearly different from the rHF group. The genetic difference between the rDF and rHF is slightly larger than that between the hDF and rHF. In the past 40 years, the genetic diversity was reduced in the DF breed and it became more homogeneous. However, the breeders managed to maintain a low level of inbreeding. Especially, inbreeding due to recent ancestors was largely absent in rDF.

Stronen et al. (2019) studied the effects of managing small unique lineages within breeds separately or managed together at breed level. What is the best strategy to avoid the risks of inbreeding and genetic drift due to low effective population sizes? They examined the genetic diversity of native and commercial cattle (*Bos taurus*) breeds, including the very small population of Danish Jutland cattle. They established the population structure and genetic diversity within breeds and lineages by genotyping 770 K SNP loci. They included older cryopreserved samples to determine whether the use of cryo-conserved semen is a real opportunity for the re-introduction of lost genetic diversity. They proved the genetic uniqueness of native domestic breeds and emphasized the need for diligent conservation plans, taking into account the unique lineages, in which inbreeding is balanced with carefully designed outcrossing. The use of cryo-conserved semen of founders can indeed support the preservation of traditional genetic characteristics of native domestic breeds.

Dechow et al. (2020) found that two Holstein Friesian bulls born in the 1950s determine the male lineage of more than 99% of all known Holstein artificial insemination (AI) cryo-conserved bulls in the United States. All Holstein bulls can be traced back to only two bulls born in the late 1800s. This means that the genetic variation for the Y chromosome in US Holstein bulls is very limited because the Y chromosome is only transmitted from sire to son. From two additional male lineages, semen was available in the USDA gene bank. Semen from bulls of those lineages was used to produce seven bulls and eight heifers by *in vitro* embryo production with oocytes from elite modern genetic females. The genomic breeding values of these seven bulls indicate that the lost lineages can be re-introduced in one generation using elite dams, resulting in a breed average genetic value for economic merit for the seven bulls. This genetic value was reached through a high genetic merit for fertility, a near-average genetic merit for fat and protein yield, and a below-average genetic merit for udder and physical conformation.

### Runs of Homozygosity as Indicators for Genetic Diversity

Before the era of genomics, there was general consensus on how to manage genetic diversity in livestock populations. Rates of pedigree-based inbreeding and kinship (ΔF and Δf) had to be limited to <1% per generation, and pedigree-based optimal contribution selection (OCS) was the method of choice to do so. Woolliams (2007) recommended that the rates of genomic inbreeding in small populations of livestock should remain below 0.5–1% per generation. Higher rates of inbreeding should be avoided. They lead to inbreeding depression through the presence of homozygous recessive alleles with negative effects and to a deterioration of traits due to the absence of favorable dominance effects at QTL, which are expressed in heterozygotes.

Nowadays, rates of genomic-based inbreeding and kinship are the characteristics used to manage diversity. They differ from pedigree based rates of inbreeding used in the past in selection schemes (Meuwissen and Oldenbroek, 2017). When data from dense marker genotyping are used to calculate rates of inbreeding, these rates include loci directly affected by selection driving allele frequency changes. The assumption for the pedigree based rate of inbreeding is that inbreeding is determined by neutral loci not linked to loci affected by selection. In reality, these unlinked loci are unlikely to exist. Thus, the realized molecular inbreeding is expected to be higher in breeding programs without an optimal contribution strategy. This is caused by within family selection of animals that get the same advantageous chromosome regions in the process of Mendelian sampling. Thus, when a pedigree based inbreeding should not be higher than 1 per cent per generation, the genomic rate of inbreeding should not be higher than 0.5 per cent (Meuwissen and Oldenbroek, 2017).

Runs of homozygosity (ROHs) are frequently used to measure inbreeding. ROHs are long stretches of two homologous chromosomes within the same individual that are identical. They are homozygous for all the loci within these stretches (Fernández and Bennewitz, 2017). ROHs reflect Identical By Descent (IBD) because it is highly unlikely that two identical long haplotypes are not copies of the same ancestral one. It is expected that a long ROH comes from a recent ancestor, and therefore it mirrors recent inbreeding. The shorter ROHs come from more distant ancestors. The proportion of the genome that is included in such an ROH is a measure of inbreeding (*F*_*ROH*_).

When a lot of stakeholders take part in the selection of parents and the number of their offspring, as it is the case in nearly all species except commercial pig and poultry breeding, mean inbreeding coefficients may fluctuate from generation to generation. Populations can suffer from inbreeding effects due to bottle necks in the past, a high selection intensity for a limited number of traits or a popular sire effect. Effects of selection on inbreeding and popular sire effects can be at stake in each generation. This implies that inbreeding rates from generation to generation should be carefully controlled in populations under selection, and in particular in rare breeds with a small effective size.

Doekes et al. (2021) used pedigree data and found that recent inbreeding caused more inbreeding depression than inbreeding from more distant ancestors. This pleads for the use of Genomic Optimal Contribution Selection (GOCS) with a relationship matrix based on long ROH segments. Rates of inbreeding and kinship require comparisons of average inbreeding coefficients between several different generations. Gene banks often contain bulls that played an important role in breeding previous generations. Their DNA can be used to determine genomic inbreeding and kinship rates.

Doublet et al. (2019) studied the effect of genomic selection on the genetic diversity of three French dairy breeds: Montbéliarde, Normande, and Holstein. Their data originated from (partly) cryo-conserved bulls born between 2005 and 2015. They calculated annual genetic gains and inbreeding rates based on runs of homozygosity (ROH) and pedigree data. They paid special attention to the mean ROH length within breeds before and after the implementation of genomic selection. No significant change in inbreeding rates was found in the two national breeds, Montbéliarde and Normande. A significant increase in inbreeding rate was calculated for the Holstein breed at 0.55% per year based on ROHs and 0.49% per year based on pedigree data. This is equal to a rate of 1.36 and 1.39% per generation, respectively. The mean ROH length was longer for the Holstein breed than for the other two breeds, due to higher levels of inbreeding in recent generations. They concluded that the annual genetic gain increased for bulls from the three major French dairy cattle breeds after the start of genomic selection. However, the massive use of a popular sire in the Holstein breed caused the increase in the mean ROH length.

Gautason et al. (2021) used 50 k genotypes of more than 8,000 Icelandic cattle, including 636 cryo-conserved bulls, to estimate the genomic and pedigree-based inbreeding using long ROHs. They also used 47 Icelandic bulls genotyped with a 770 k SNP to compare them with other Nordic dairy cattle breeds. Average inbreeding coefficients according to pedigree and ROHs were 0.0621 and 0.101, respectively. They also computed ROH-based autosomal inbreeding coefficients. No severe historical inbreeding was found. The effective population sizes for the years 2009–2017 according to pedigree, ROHs, genomic relationship matrix, excess of homozygosity and individual increase in inbreeding were 81, 65, 60, 58, and 92, respectively. They concluded that inbreeding rates in Icelandic cattle are in line with FAO guidelines.

Doekes et al. (2018b) evaluated genome-wide and region-specific genetic diversity and inbreeding in the Dutch-Flemish Holstein Friesian (HF) selection scheme. In recent decades this scheme changed drastically. This implies the introduction of optimal contribution selection (OCS; around 2000), a major change in the composition of the breeding goal (around 2000), and the implementation of genomic selection (GS; around 2010). Pedigree and genotype data (∼75.5 k SNPs) of 6,280 cryo-conserved AI-bulls were used to estimate rates of genome-wide inbreeding and kinship used to calculate the effective population sizes. Region-specific inbreeding trends were evaluated using ROHs. The effective population size between 1986 and 2015 varied between 69 and 102. Two major divisions were established in the genome-wide inbreeding and kinship trends. Around 2000, the inbreeding and kinship levels temporarily decreased. After the introduction of genomic selection from 2010 onwards, they sharply increased, with pedigree-based, ROH-based and marker-based inbreeding rates reaching levels of 1.8, 2.1 and 2.8% per generation, respectively. Across the genome, a substantial variation in the accumulation of inbreeding was found.

Meyermans et al. (2021) studied the genetic diversity in two populations of Belgian dual-purpose red cattle breeds. They are threatened because Belgian farmers nowadays prefer more specialized cattle breeds. In total, 270 animals, including 58 cryo-conserved bulls, of the Belgian Red and Belgian White Red cattle were genotyped with medium density SNP arrays. Genetic diversity parameters were: runs of homozygosity, effective population size, and genetic distances (Fst). ROH-based genomic inbreeding coefficients were estimated at 7.0% for Belgian Red and 6.1% for Belgian White Red cattle. The two populations had a low effective population size of 68 and 86, respectively. This illustrates the threat to their existence.

In conclusion, estimations of genetic relationships in small populations carrying rare alleles or carrying alleles rare in the larger breeds should be carried out based on WGS data. When the economic value of traits is changed in a breeding program, gene bank sires may be of value to realize such breeding goals. They can also be used to re-introduce parts of the genomes of lost founders in less controlled breeding programs. ROH’s measure clearly the effects of selection and genetic drift (although often confounded) on inbreeding at the level of the whole genome as well on parts of it. These effects are often transferred into effective population sizes to illustrate better the course of the genetic diversity in the population.

## The Improvement of the Performance and the Genetic Diversity of Living Populations

### Construction of Reference Populations for Breeds

Knowledge of pedigrees is the first prerequisite to starting a breeding program. But not all farmers are participants in official pedigree recording programs. Small populations of rare breeds might benefit a lot when the population size can be extended because this can prevent inbreeding. Anecdotal and phenotypic information can indicate that animals belong to a certain breed, but breeding organizations have to be sure of that before these animals can fulfill a role in their breeding program. Genomics gives the opportunity to construct a reference population of individuals whose breed of origin is recorded over several generations. Gene bank sires always have an official registered pedigree and their DNA-composition may offer an important contribution to such a reference population for their breed. It is important that the reference population comprises the total genetic diversity of the breed at stake. The SNP markers can be selected from that “complete” reference population to be able to discriminate accurately amongst the breeds.

Hulsegge et al. (2019b) constructed reference populations for the Dutch cattle breeds partly based on SNP markers of gene bank sires. For the purity test, they used a threshold value equal or higher than 0.775 for which an non-registered animal is assigned to a breed. Out of tens of thousands of SNP markers, only 133 SNPs were needed to assign animals correctly to Dutch cattle breeds. Crossbred animals and animals from foreign breeds were identified as well.

Gebrehiwot et al. (2021) developed small SNP panels that accurately estimate the total proportion of dairy breeds and determine the parents of individuals in West and East African crossbred dairy cows. In the African smallholder system, pedigrees are not officially recorded, and often crossbreeding with dairy breeds is at stake. The identified low-cost SNP assays could complete the pedigree records in African smallholder systems. They facilitate effective breeding decisions to breed animals with the desired composition of the breeds available.

### Construction of Reference Populations for Genomic Selection

In the genomic era, the genomic prediction of breeding values is an important, but challenging opportunity for the programs of small local breeds (Meuwissen and Oldenbroek, 2017). It offers the opportunity to increase the performance of breeds. Genomic prediction is based on large haplotype blocks created by family structure and a small effective population size. These haplotype blocks may contain several QTL. The effects of the alleles of the individual QTL are confounded. But the combination of many small local breeds with SNP and phenotypic data in the genomic selection scheme offers a large variety of haplotype blocks that can be used for genomic prediction. Then, the effects of individual QTL, present in several breeds, may be untangled. The knowledge of haplotypes and QTLs facilitates choosing the animals for conservation.

In small local populations, it is often impossible to create a reference population of sufficient size. Then data from animals of other breeds has to be added before the genomic prediction can start (Hozé et al., 2014). But, animals from the current population have a much higher reference population value than animals from other populations. Hence, it is important to create as many as possible reference animals from the current population. Males and females with phenotypic and SNP data are relevant. Cryo-conserved sires with phenotypic data can enlarge the reference populations and make genetic relationships in the reference population and between the reference and the “test” population of young animals and embryos more accurate. The latter is important because small populations need to use all opportunities to achieve accurate genomic predictions. When variable selection genomic prediction methods are used, the across and within breed genomic predictions can be carried out effectively (Kemper et al., 2015). The incorporation of reference populations from breeds that are related to the current breed is to be preferred (Lund et al., 2014). However, the across breed reference populations need to be significantly larger than the reference populations for a single breed (Wientjes, 2016).

Marjanovic et al. (2020) studied the possibilities for genomic selection in red dairy breeds, based on genomic and phenotypic data of cryo-conserved sires. The different breed-specific reference populations were all too small for accurate genomic prediction. Therefore, they studied the effect of adding individuals from another breed. The effective number of chromosome segments (*Me*) was used to estimate the relatedness between individuals from different breeds. It can also be used to prioritize breeds for conservation. The *Me* is also used an important parameter for the accuracy of genomic prediction. The *Me* can be estimated both within a population and between two populations or breeds. It is expressed as the reciprocal of the variance of genomic relationships. The six red Dutch rare breeds indicated a high variability of *Me*. Between breeds, the *Me* ranges from ∼3,500 to ∼17,400. It indicates the levels of relatedness between the breeds were different. Three clusters of breeds were found: 1) the MRY, Deep Red, and Improved Red; 2) the Dutch Friesian and Dutch Belted; and 3) the Groningen White Headed. The relatedness between breeds within the first two clusters is high. However, across-breed genomic prediction is still hampered due to the low number of genotyped individuals. An increase in this number is very effective. It appeared that for each of the six breeds, 11–133 reference animals from other breeds are needed to achieve the same accuracy of genomic prediction as an additional individual from the same breed.

### Methods for Introgression of Traits

Sometimes a gene bank contains animals with a trait that is not present in a living population, but that trait can have a high value for that population. Then, introgression of the desired allele(s) responsible for that trait in the donor animals into the recipient population can be considered (Meuwissen and Oldenbroek, 2017). Here, two methods for introgression from a donor to a recipient breed will be discussed: 1) the transfer of an allele or 2) the transfer of set of alleles. The two can be realized by crossing parents from the donor breed to the recipient breed, followed by systematic backcrossing with the recipient breed. In each generation, parents for the next generation are chosen who carry the desired allele(s). The donor breed may be a small local breed that contains a desirable trait. The recipient maybe a mainstream breed which is lacking the desired trait. Further it is assumed that the allele(s) coding for the desired trait is (are) known, or narrow QTL regions with the allele(s) are known. This is known as Marker Assisted Introgression (MAI). It is a useful tool to introduce traits from a (conserved) donor population into a (mainstream) recipient population. A short generation interval facilitates this process. It is only worthwhile for a trait that has indeed a high value for the recipient breed. Hence, this value should compensate for the loss of several generations of selection in the mainstream breed because the donor breed often has lower performance.

Genomic introgression (GI) is a method to introgress a trait from a cryo-conserved donor population into a mainstream recipient population in the case of a complex trait. The genetic architecture of the trait is unidentified, for example, the architecture is complex and many genes determine the trait (Meuwissen and Oldenbroek, 2017). Applying GI, the first step is to produce crossbred offspring from donor and recipient animals. This will increase the genetic variance for the trait of interest in the offspring. The second step is a genomic selection program to improve the total performance. The weight of the trait of interest in the selection program is sufficient to obtain a positive selection response. The animals from the donor breed usually have lower performance than the recipient breed. Therefore, the higher variance in the crossbred generation is used to create higher genetic progress in subsequent generations.

Historically, introgression has been used to upgrade and to improve an important trait in breeds. The FecB fecundity QTL is first found in Australian Booroola sheep. This QTL, improving the litter size significantly, has been introgressed into a large number of other breeds (Fogarty, 2009).

Polledness in cattle has received increasing interest in recent years for welfare reasons. It is based on two different dominant alleles that are situated very close together in the cattle genome (Allais-Bonnet et al., 2013). In the dominating mainstream dairy breeds, polledness was absent. They are found in smaller breeds, especially in northern Europe (Cozzi et al., 2015), where they are fixed or segregating. Cryo-collections of breeds may contain the relevant alleles. Genomics can identify the carriers, which can be used for introgression. But, in many breeds where polledness was never observed, it is unlikely to detect carriers in cryo-conserved semen (Windig et al., 2015).

### Methods for Removing Unwanted Introgression of Foreign Breeds

Many breeds have experienced migration or introgression in the past. Herdbooks often want or have to register only purebred animals. By selecting animals that minimize genomic co-ancestry between current animals and animals carrying the introgressed parts of the genome, the original genetic background of a breed can be recovered. With genomic information, this expires more efficiently than with pedigree information (Amador et al., 2013; Amador et al., 2014).

Kohl et al. (2020) found that applying standard Optimum Contribution Selection methodology in small local breeds with historical introgression could lead to a more intense selection of introgressed genetic material. The reason is that the introgressed alleles improve the rate of genetic gain and reduce the average kinship as an outcome of OCS. Consequently, small local breeds become extinct. In a simulation study, they used the advanced OCS (aOCS) approach that takes into account the introgressed genetic material. They created populations from the historical gene pool by using aOCS and took care that the simulated populations were comparable with real data. Historical breeding decisions that favored introgressed material could have been avoided by using aOCS. The genetic gain would have been at least 12.2% lower. However, the presence of introgressed genetic material, the genetic diversity, and native genetic diversity would have been more satisfactory for a small local breed whose breed purity should be enhanced.

Wang et al. (2017) found In the German Angler and Vorderwald cattle, a significant positive correlation between Migrant Contributions (MC) and estimated breeding values of the selection candidates. This means that traditional OCS would increase MC. They included MC in OCS and modified the kinships that account for the breed origin of alleles. Three OCS alternatives were simulated, taking into account minimizing kinship, minimizing MC, and maximizing genetic gain in the offspring generation. In the simulations, the inbreeding rate should not become higher than 1%, and at least 30% of the maximum progress should be achieved for all other criteria. In the traditional OCS (reference scenario), the highest breeding values were found with the restriction of classical kinship. In this case, the magnitude of MC in the progeny generation was not in control. When constraining or minimizing MC, the kinship of native alleles increased compared to the reference scenario. They concluded that including MC and kinship at native alleles is necessary when you want to maintain the genetic originality and the diversity of native alleles in breeding program aiming at genetic gain and control of inbreeding.

Schaler et al. (2018) studied the possibilities of reverse introgression in two local red cattle breeds from Northern Germany. They had pedigree data for 90,783 individuals for the German Angler breed and 187,255 individuals for the Red Dual-Purpose cattle breed. Information on sex, date of birth, breed percentage, and conventional breeding values was available. The native genetic contribution of individuals could be included as an additional trait in the total merit index as an attempt to recover a part of the native genetic background. Marker information that accounts for Mendelian sampling improved the native contributions. The maintenance of a sufficient genetic diversity of native alleles needs an advanced OCS with proper constraints.

In conclusion, gene bank sires have well recorded pedigrees and are used to construct reference populations to test breed purity. In combination with their breeding values for performance traits, they can strengthen the relationships in populations needed in genomic selection schemes. Methods for the introgression of traits from gene bank sires into living populations are well developed and effective. Optimum contribution methodology is available and is used to recover the native genetic background of breeds in which crossbreeding took place in the past.

## Overall Conclusion

Gene banks can be considered as a sustainable and reliable archive of genetic diversity. Genomics gives three relevant stakeholders important tools to improve their efforts to conserve and use genetic diversity: 1) Gene bank management has the opportunity to use genomics to prioritize breeds and lines and animals within breeds for conservation and to optimize the collections. They may facilitate the creation of reference populations to test animals for breed purity and for genomic selection. At request, they can provide straws for genomic research and for the activities of rare breed associations and commercial companies. 2) rare breed associations have the opportunity to use genomics to add non-recorded animals to their populations, to re-use sires whose genomes are no longer present in the populations, to monitor the relationship and inbreeding over generations and to remove parts of the genome of other introgressed breeds, and 3) commercial breeding companies have the opportunity to use the genotypic and phenotypic data of gene bank sires into their reference populations for genomic selection, may re-use sires when afterwards it reveals that parts of their genome are interesting and no longer present in their current breeding sires, can monitor kinship and inbreeding over generations and can consider the introgression of interesting genes in gene bank sires not present in their current breeding animals.

## References

[B1] Allais-Bonnet A. Grohs C. Medugorac I. Krebs S. Djari A. Graf A. (2013). Novel Insights into the Bovine Polled Phenotype and Horn Ontogenesis in Bovidae. PLoS ONE 8 (5), e63512. 10.1371/journal.pone.0063512 PubMed Abstract | 10.1371/journal.pone.0063512 | Google Scholar 23717440 PMC3661542

[B2] Amador C. Fernández J. Meuwissen T. H. (2013). Advantages of Using Molecular Coancestry in the Removal of Introgressed Genetic Material. Genet. Sel. Evol. 45, 13. 10.1186/1297-9686-45-13 PubMed Abstract | 10.1186/1297-9686-45-13 | Google Scholar 23634969 PMC3652759

[B3] Amador C. Hayes B. J. Daetwyler H. D. (2014). Genomic Selection for Recovery of Original Genetic Background from Hybrids of Endangered and Common Breeds. Evol. Appl. 7, 227–237. 10.1111/eva.12113 PubMed Abstract | 10.1111/eva.12113 | Google Scholar 24567744 PMC3927885

[B4] Bennewitz J. Eding H. Ruane J. Simianer H. (2007). “Chapter 6: Selecting Breeds for Conservation,” in Utilisation and Conservation of Farm Animal Genetic Resources. Editor Oldenbroek K. (Wageningen: Academic Publishers). ISBN 978-90-8686-032-6. Google Scholar

[B5] Berg P. Windig J. J. (2017). “Chapter 6: Management of Cryo-Collections with Genomic Tools,” in Genomic Management of Animal Genetic Diversity (Netherlands: Wageningen Academic Publishers). ISBN: 978-90-8686-297-9. Google Scholar

[B6] Blackburn H. D. Wilson C. S. Krehbiel B. (2019). Conservation and Utilization of Livestock Genetic Diversity in the United States of America through Gene Banking. Diversity 11 (12), 244. 10.3390/d11120244 10.3390/d11120244 | Google Scholar

[B7] Boitard S. Paris C. Sevane N. Servin B. Bazi-Kabbaj K. Dunner S. (2021). Gene banks as reservoirs to detect recent selection: the example of the Asturiana de los Valles Bovine breed. Front. Genet. 12, 575405. 10.3389/fgene.2021.575405 PubMed Abstract | 10.3389/fgene.2021.575405 | Google Scholar 33633776 PMC7901938

[B8] Cozzi G. Gottardo F. Brscic M. Contiero B. Irrgang N. Knierim U. (2015). Dehorning of Cattle in the EU Member States: A Quantitative Survey of the Current Practices. Livest. Sci. 179, 4–11. 10.1016/j.livsci.2015.05.011 10.1016/j.livsci.2015.05.011 | Google Scholar

[B9] Dechow C. D. Liu W. S. Specht L. W. Blackburn H. (2020). Reconstitution and Modernization of Lost Holstein Male Lineages Using Samples from a Gene Bank. J. Dairy Sci. 103, 4510–4516. 10.3168/jds.2019-17753 PubMed Abstract | 10.3168/jds.2019-17753 | Google Scholar 32171516

[B10] Doekes H. P. Veerkamp R. F. Bijma P. Hiemstra S. J. Windig J. J. (2018a). Value of the Dutch Holstein Friesian Germplasm Collection to Increase Genetic Diversity and Improve Genetic Merit. J. Dairy Sci. 101, 10022. 10.3168/jds.2018-15217 10.3168/jds.2018-15217 | Google Scholar 30219429

[B11] Doekes H. P. Veerkamp R. F. Bijma P. Hiemstra S. J. Windig J. J. (2018b). Trends in Genome-wide and Region-specific Genetic Diversity in the Dutch-Flemish Holstein-Friesian Breeding Program from 1986 to 2015. Genet. Sel. Evol. 50, 15. 10.1186/s12711-018-0385-y PubMed Abstract | 10.1186/s12711-018-0385-y | Google Scholar 29642838 PMC5896142

[B12] Doekes H. P. Bijma P. Windig J. J. (2021). How Depressing Is Inbreeding? A Meta-Analysis of 30 Years of Research on the Effects of Inbreeding in Livestock. Genes 12, 926. 10.3390/genes12060926 PubMed Abstract | 10.3390/genes12060926 | Google Scholar 34207101 PMC8234567

[B13] Doekes H. P. (2020). Genomic Characterization and Conservation of Genetic Diversity in Cattle. PhD thesis. Wageningen, Netherlands: Wageningen University, 216. ISBN: 978-94-6395-423-5. 10.18174/523321 10.18174/523321 | Google Scholar

[B14] Doublet A.-C. Croiseau P. Fritz S. Michenet A. Hozé C. Danchin-Burge C. (2019). The Impact of Genomic Selection on Genetic Diversity and Genetic Gain in Three French Dairy Cattle Breeds. Genet. Sel. Evol. 51, 52. 10.1186/s12711-019-0495-1 PubMed Abstract | 10.1186/s12711-019-0495-1 | Google Scholar 31547802 PMC6757367

[B15] Eding H. Crooijmans R. P. Groenen M. A. Meuwissen T. H. (2002). Assessing the Contribution of Breeds to Genetic Diversity in Conservation Schemes. Genet. Sel. Evol. 34, 613–633. 10.1186/1297-9686-34-5-613 PubMed Abstract | 10.1186/1297-9686-34-5-613 | Google Scholar 12427389 PMC2705437

[B16] Engelsma K. A. Veerkamp R. F. Calus M. P. L. Windig J. J. (2014). Consequences for Diversity when Animals are Prioritized for Conservation of the Whole Genome or of One Specific Allele. J. Anim. Breed. Genet. 131, 61–70. 10.1111/jbg.12052 PubMed Abstract | 10.1111/jbg.12052 | Google Scholar 25099790

[B17] Eynard S. E. Windig J. J. Leroy G. van Binsbergen R. Calus M. (2015). The Effect of Rare Alleles on Estimated Genomic Relationships from Whole Genome Sequence Data. BMC Genet. 16, 24. 10.1186/s12863-015-0185-0 PubMed Abstract | 10.1186/s12863-015-0185-0 | Google Scholar 25887220 PMC4365517

[B18] Eynard S. E. Windig J. J. Hiemstra S. J. Calus M. P. L. (2016). Whole-genome Sequence Data Uncover Loss of Genetic Diversity Due to Selection. Genet. Sel. Evol. 48, 33. 10.1186/s12711-016-0210-4 PubMed Abstract | 10.1186/s12711-016-0210-4 | Google Scholar 27080121 PMC4831198

[B19] FAO (2012). Cryoconservation of Animal Genetic Resources. Rome: FAO Animal Production and Health. Guidelines No. 12. Google Scholar

[B20] Fernández J. Bennewitz J. (2017). “Chapter 2: Defining Genetic Diversity Based on Genomic Tools,” in Genomic Management of Animal Genetic Diversity (Netherlands: Wageningen Academic Publishers). ISBN: 978-90-8686-297-9. Google Scholar

[B21] Fogarty N. M. (2009). A Review of the Effects of the Booroola Gene (FecB) on Sheep Production. Small Ruminant Res. 85, 75–84. 10.1016/j.smallrumres.2009.08.003 10.1016/j.smallrumres.2009.08.003 | Google Scholar

[B22] Gautason E. Schönherz A. A. Sahana G. Guldbrandtsen B. (2020). Relationship of Icelandic Cattle with Northern and Western European Cattle Breeds, Admixture and Population Structure. Acta Agric. Scand. Sect. A Animal Sci. 69 (1-2), 25–38. 10.1080/09064702.2019.1699951 10.1080/09064702.2019.1699951 | Google Scholar

[B23] Gautason E. Schönherz A. A. Sahana G. Guldbrandtsen B. (2021). Genomic Inbreeding and Selection Signatures in the Local Dairy Breed Icelandic Cattle. Anim. Genet. 52, 251–262. 10.1111/age.13058 PubMed Abstract | 10.1111/age.13058 | Google Scholar 33829515

[B24] Gebrehiwot N. Z. Strucken E. M. Marshall K. Aliloo H. Gibson J. P. (2021). SNP Panels for the Estimation of Dairy Breed Proportion and Parentage Assignment in African Crossbred Dairy Cattle. Genet. Sel. Evol. 53, 21. 10.1186/s12711-021-00615-4 PubMed Abstract | 10.1186/s12711-021-00615-4 | Google Scholar 33653262 PMC7923343

[B25] Hozé C. Fritz S. Phocas F. Boichard D. Ducrocq V. Croiseau P. (2014). Efficiency of Multi-Breed Genomic Selection for Dairy Cattle Breeds with Different Sizes of Reference Population. J. Dairy. Sci. 97, 3918–3929. 10.3168/jds.2013-7761 PubMed Abstract | 10.3168/jds.2013-7761 | Google Scholar 24704232

[B26] Hulsegge B. Calus M. P. L. Oldenbroek J. K. Windig J. J. (2016). Conservation Priorities for the Different Lines of Dutch Red and White Friesian Cattle Change when Relationships with Other Breeds are Taken into Account. J. Anim. Breed. Genet. 134, 69–77. 10.1111/jbg.12233 PubMed Abstract | 10.1111/jbg.12233 | Google Scholar 27461414

[B27] Hulsegge I. Calus M. Hoving-Bolink R. Lopes M. Megens H.-J. Oldenbroek K. (2019a). Impact of Merging Commercial Breeding Lines on the Genetic Diversity of Landrace Pigs. Genet. Sel. Evol. 51, 60. 10.1186/s12711-019-0502-6 PubMed Abstract | 10.1186/s12711-019-0502-6 | Google Scholar 31664893 PMC6819590

[B28] Hulsegge I. Schoon M. Windig J. Neuteboom M. Hiemstra S. J. Schurink A. (2019b). Development of a Genetic Tool for Determining Breed Purity of Cattle. Livest. Sci. 223, 60–67. 10.1016/j.livsci.2019.03.002 10.1016/j.livsci.2019.03.002 | Google Scholar

[B29] Hulsegge I. Oldenbroek K. Bouwman A. Veerkamp R. Windig J. (2022). Selection and Drift: A Comparison between Historic and Recent Dutch Friesian Cattle and Recent Holstein Friesian Using WGS Data. Animals 12, 329. 10.3390/ani12030329 PubMed Abstract | 10.3390/ani12030329 | Google Scholar 35158654 PMC8833835

[B30] Huson H. J. Sonstegard T. S. Godfrey J. Hambrook D. Wolfe C. Wiggans G. (2020). A Genetic Investigation of Island Jersey Cattle, the Foundation of the Jersey Breed: Comparing Population Structure and Selection to Guernsey, Holstein, and United States Jersey Cattle. Front. Genet. 11, 366. 10.3389/fgene.2020.00366 PubMed Abstract | 10.3389/fgene.2020.00366 | Google Scholar 32362912 PMC7181675

[B53] Kemper K.E. Reich C. M. Bowman P. J. van der Jagt C. J. Chamberlain A. J. Mason B. A. (2015). Improved Precision of QTL Mapping Using a Nonlinear Bayesian Method in a Multi-Breed Populations Leads to a Greater Accuracy of Across-Breed Genomic Predictions. Genetics Selection Evolution 47, 29. 10.1186/s12711-014-0074-4 | Google Scholar PMC4399226 25887988

[B31] Kohl S. Wellmann R. Herold P. (2020). Implementation of Advanced Optimum Contribution Selection in Small-Scale Breeding Schemes: Prospects and Challenges in Vorderwald Cattle. Animal 14 (3), 452–463. 10.1017/S1751731119002295 PubMed Abstract | 10.1017/S1751731119002295 | Google Scholar 31597583 PMC7026723

[B32] Leroy G. Besbes B. Boettcher P. Hoffmann I. Capitan A. Baumung R. (2015). Rare Phenotypes in Domestic Animals: Unique Resources for Multiple Applications. Anim. Genet. 47, 141–153. 10.1111/age.12393 PubMed Abstract | 10.1111/age.12393 | Google Scholar 26662214

[B33] Lund M. S. Su G. Janss L. Guldbrandtsen B. Brøndum R. F. (2014). Genomic Evaluation of Cattle in a Multi-Breed Context. Livest. Sci. 166, 101–110. 10.1016/j.livsci.2014.05.008 10.1016/j.livsci.2014.05.008 | Google Scholar

[B34] Marjanovic J. Hulsegge B. Calus M. (2020). Relatedness between Numerically Small Dutch Red Dairy Cattle Populations and Possibilities for Multibreed Genomic Prediction. J. Dairy Sci. 104, 4498–4506. 10.3168/jds.2020-19573 10.3168/jds.2020-19573 | Google Scholar 33551169

[B35] Meuwissen T. H. E. Oldenbroek J. K. (2017). “Chapter 5: Management of Genetic Diversity Including Genomic Selection in Small *In Vivo* Populations,” in Genomic Management of Animal Genetic Diversity (Netherlands: Wageningen Academic Publishers). ISBN: 978-90-8686-297-9. Google Scholar

[B36] Meyermans R. Gorssen W. Buys N. Janssens S. (2021). Genomics Confirm an Alarming Status of the Genetic Diversity of Belgian Red and Belgian White Red Cattle. Animals 11, 3574. 10.3390/ani11123574 PubMed Abstract | 10.3390/ani11123574 | Google Scholar 34944349 PMC8697887

[B37] Oldenbroek J. K. (1999). “Chapter 1: Introduction,” in Genebanks and the Conservation of Farm Animal Genetic Resources (Lelystad, Netherlands: DLO-Institute for Animal Science and Health). ISBN: 90-75124-06-6. Google Scholar

[B38] Oldenbroek J. K. (2007). “Chapter 1: Introduction,” in Utilisation and Conservation of Farm Animal Genetic Resources (Netherlands: Wageningen Academic Publishers). ISBN 978-90-8686-032-6. Google Scholar

[B39] Oldenbroek J. K. (2017). Genomic Management of Animal Genetic Diversity. Netherlands: Wageningen Academic Publishers. ISBN: 978-90-8686-297-9. Google Scholar

[B40] Passemard A. S. Hiemstra S. J. Tixier-Boichard M. Danchin-Burge C. (2018). “Mapping the Diversity and Characteristics of European Farm Animal Genetic Collections: Banks or Museums?,” in Proceedings of the World Congress on Genetics Applied to Livestock Production, Auckland, New Zealand, February 7–11, 2018 (Auckland, New Zealand). Google Scholar

[B41] Schaler J. Wellmann R. Bennewitz J. Thaller G. Hinrichs D. (2018). Genetic Diversity and Historic Introgression in German Angler and Red Dual Purpose Cattle and Possibilities to Reverse Introgression. Acta Agric. Scand. Sect. A Animal Sci. 68 (2), 63–72. 10.1080/09064702.2019.1600011 10.1080/09064702.2019.1600011 | Google Scholar

[B42] Schmidtmann C. Schönherz A. Guldbrandtsen B. Marjanovic J. Calus M. HinrichsThaller D. G. (2021). Assessing the Genetic Background and Genomic Relatedness of Red Cattle Populations Originating from Northern Europe. Genet. Sel. Evol. 53, 23. 10.1186/s12711-021-00613-6 PubMed Abstract | 10.1186/s12711-021-00613-6 | Google Scholar 33676402 PMC7936461

[B43] Signer-Hasler H. Burren A. Neuditschko M. Frischknecht M. Garrick D. Stricker C. (2017). Population Structure and Genomic Inbreeding in Nine Swiss Dairy Cattle Populations. Genet. Sel. Evol. 49, 83. 10.1186/s12711-017-0358-6 PubMed Abstract | 10.1186/s12711-017-0358-6 | Google Scholar 29115934 PMC5674839

[B44] Smith C. (1984). Genetic Aspects of Conservation in Farm Livestock. Livest. Prod. Sci. 11, 37–48. 10.1016/0301-6226(84)90005-8 10.1016/0301-6226(84)90005-8 | Google Scholar

[B45] Stronen A. V. Pertoldi C. Iacolina L. Kadarmideen H. N. Kristensen T. N. (2019). Genomic Analyses Suggest Adaptive Differentiation of Northern European Native Cattle Breeds. Evol. Appl. 12, 1096–1113. 10.1111/eva.12783 PubMed Abstract | 10.1111/eva.12783 | Google Scholar 31293626 PMC6597895

[B46] Upadhyay M. Eriksson S. Mikko S. Strandberg E. Stålhammar H. Groenen M. A. M. (2019). Genomic Relatedness and Diversity of Swedish Native Cattle Breeds. Genet. Sel. Evol. 51, 56. 10.1186/s12711-019-0496-0 PubMed Abstract | 10.1186/s12711-019-0496-0 | Google Scholar 31578144 PMC6775670

[B47] Van Breukelen A. E. Doekes H. P. Windig J. J. Oldenbroek K. (2019). Characterization of Genetic Diversity Conserved in the Gene Bank for Dutch Cattle Breeds. Diversity 11, 229–313. 10.3390/d11120229 10.3390/d11120229 | Google Scholar

[B48] Wang Y. Bennewitz J. Wellmann R. (2017). Novel Optimum Contribution Selection Methods Accounting for Conflicting Objectives in Breeding Programs for Livestock Breeds with Historical Migration. Genet. Sel. Evol. 49, 45. 10.1186/s12711-017-0320-7 PubMed Abstract | 10.1186/s12711-017-0320-7 | Google Scholar 28499352 PMC5427594

[B49] Wellmann R. Bennewitz J. (2019). Key Genetic Parameters for Population Management. Front. Genet. 10, 667. 10.3389/fgene.2019.00667 PubMed Abstract | 10.3389/fgene.2019.00667 | Google Scholar 31475027 PMC6707806

[B50] Wientjes Y. C. J. (2016). Multi-population Genomic Prediction. PhD Thesis. Netherlands: Wageningen University, 268. Google Scholar

[B51] Windig J. J. Hoving-Bolink R. A. Veerkamp R. F. (2015). Breeding for Polledness in Holstein Cattle. Livest. Sci. 179, 96–101. 10.1016/j.livsci.2015.05.021 10.1016/j.livsci.2015.05.021 | Google Scholar

[B52] Woolliams J. (2007). “Chapter 7: Genetic Contributions and Inbreeding,” in Utilisation and Conservation of Farm Animal Genetic Resources. Editor Oldenbroek K. (Netherlands: Wageningen Academic Publishers). ISBN 978-90-8686-032-6. Google Scholar

